# Internal dislocation of scapula following thoracotomy for lung transplantation – a case report

**DOI:** 10.1186/1755-7682-2-10

**Published:** 2009-04-16

**Authors:** V Palissery, T Veenith, NB Siddaiah, L Brennan

**Affiliations:** 1 Norfolk and Norwich University Hospitals NHS Trust, Norwich, UK; 2 Papworth Hospitals NHS foundation trust, Papworth Everard, Cambridge, UK; 3 Addenbrookes hospital Cambridge, Hills road, Cambridge, UK

## Abstract

**Introduction:**

The diagnosis of chest pain at times can be challenging and requires a detailed history, thorough physical examination and investigations including imaging in post transplantation patients. This case is an example of a rare cause of pleuritic chest pain, which was initially misdiagnosed as a haemothorax. The correct diagnosis of dislocated scapula was delayed for three days resulting in considerable discomfort for the patient.

**Case presentation:**

We present a case of dislocation of scapula following thoracotomy for single lung transplantation. This complication should be considered in the list of differential diagnosis for the pleuritic chest pain after thoracotomy.

**Conclusion:**

This case highlights the importance of careful positioning of the patient perioperatively and when they are sedated and ventilated after the surgical procedure.

## Introduction

The number of patients undergoing lung transplantation globally has averaged between 1300 – 1400 per year and patients currently receiving a lung transplantation can be expected to have survival rates of approximately 75% at 1 year and 45% at 5 years from the time of transplantation [1]. Life-threatening complications of lung transplantation occur in 37% of the cases, with a mortality of 13% within first three months after the transplantation [2]. Post-operative deaths are commonly due to bleeding, sepsis, acute rejection, adult respiratory distress syndrome, multiorgan failure, diffuse alveolar damage and respiratory failure [2]. Other complications include mechanical pulmonary complications, trauma to adjacent structures, bleeding, infection, reperfusion oedema and rejection. [3-5]. We present a rare complication of thoracotomy namely, dislocation of the scapula presenting as pleuritic chest pain for which the diagnosis was delayed for three days.

## Case report

**Figure 1:**
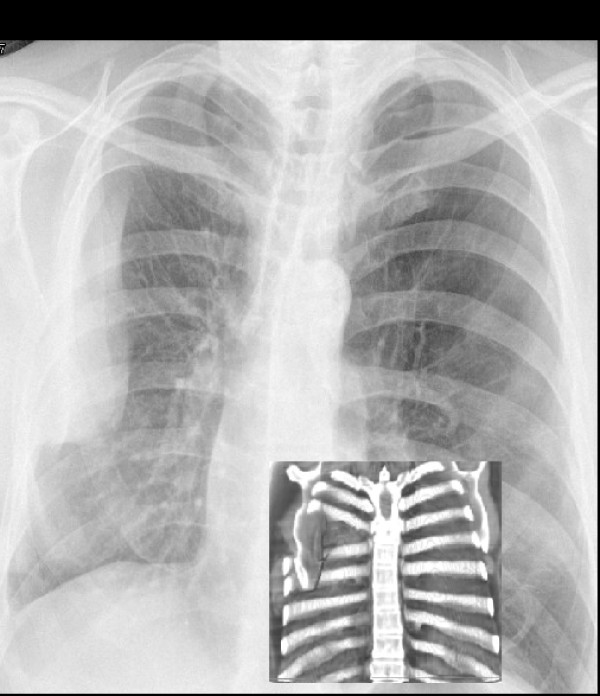
**Chest radiograph showing a right peripheral density following dislocation of the scapula**.

A 64-year-old man underwent single lung transplantation via a right thoracotomy incision in the 5th intercostal space. Post-operatively, after one month he underwent routine check bronchoscopy and transbronchial biopsy utilising with propofol and suxamethonium for general anaesthesia. Following this procedure he complained of right-sided pleuritic chest pain whilst in the recovery room. Post procedure observations were within normal limits. Clinical assessment confirmed a pleuritic chest pain which was worsened by worsened by abduction and external rotation of shoulder. Chest radiograph was performed to exclude haemopneumothorax as a complication of transbronchial biopsy. This revealed a hyperdense shadow over the lateral aspect of right chest wall with an increase in intercostal distance between the 5th and 6th ribs which was thought to be a haemothorax (Fig. 1).

**Figure 2:**
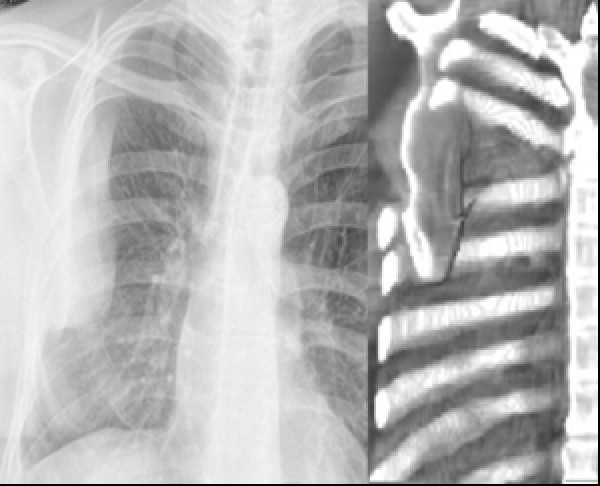
**Computer reconstructions of CT scan of chest and chest x-ray showing internal dislocation of the scapula through the thoracotomy incision**.

Because of the suspicion of a intrapleural haematoma, a diagnostic plain CT scan of chest was performed (Fig. 2). This revealed a prolapsed inferior angle of the scapula through the 5th and 6th intercostal space. In retrospect the shadow of the plain film did conform to the adjacent scapular outline.

The scapula was initially reduced by external manipulation, but the dislocation proved to be unstable and required an operative reduction. At the same operation the right thoracotomy defect between the 5^th ^and 6^th ^intercostal spaces was repaired as well.

## Discussion

Scapular dislocation is rare complication [6,7] following thoracotomy, and there should be a high index of suspicion for investigating and diagnosing this condition when the clinician is presented with pain and asymmetry following the transplantation. History, physical examination, and chest radiography are recommended for patients with pleuritic chest pain [8].

The chest x-ray appearance of a well demarcated homogenous shadow was confirmed as the dislocated scapula on CT scan reconstruction. Internal scapular dislocation into the thoracic cavity through the defect in thoracotomy incision was a complication presumably occurred in the perioperative period. As the problem presented with a pleuritic chest pain, a common problem encountered after the thoracotomy there was a delay in confirming the correct diagnosis. This adds to the list of complications due to lateral positioning for thoracotomy and post-thoracotomy care that include caval compression, ventilation-perfusion mismatch leading to oxygen desaturation, pressure injuries, muscular and ligamentous sprain, and ocular injuries [9].

This necessitated him to undergo one more anaesthetic until it was fixed by open reduction and surgical repair of thoracotomy defect. Even though this is a rare complication following thoracotomy, vigilance is advised in patients complaining of persistent chest pain in the absence of other symptoms and signs of respiratory disease.

## Consent

Written informed consent was obtained from the patient for publication of this case report and accompanying images. A copy of the written consent is available for review by the Editor-in-Chief of this journal.

## Competing interests

The authors declare that they have no competing interests.

## Authors' contributions

VP was involved in the direct care of this patient, along with TV, NS, LB wrote the manuscript. All authors has read and approved the manuscript. We thank the radiology department for providing the reconstructed images.

## References

[B1] Hosenpud JD, Bennett LE, Keck BM, Boucek MM, Novick RJ (2001). The Registry of the International Society for Heart and Lung Transplantation: eighteenth Official Report – 2001. J Heart Lung Transplant.

[B2] Collins J, Kuhlman JE, Love RB (1998). Acute, life-threatening complications of lung transplantation. Radiographics.

[B3] Leo F, Venissac N, Pop D, Anziani M, Leon ME, Mouroux J (2006). Anticipating pulmonary complications after thoractomy: the FLAM Score. J of Cardiothor Surg.

[B4] Cremer J, Hirt SW, Hein M, Heidi Böttcher (2001). Surgical Technique and Complications in Lung Transplantation. Acta Chirurgica Austriaca.

[B5] Herman SJ, Rappaport DC, Weisbrod GL, Olscamp GC, Patterson GA, Cooper JD (1989). Single lung transplantation: imaging features. Radiology.

[B6] Murray JG, McAdams HP, Erasmus JJ, Patz EF, Tapson V (1996). Complications of lung transplantation: radiologic findings. AJR Am J Roentgenol.

[B7] Gilkeson RC (1998). Intrathoracic scapula: an unusual complication of thoracotomy. AJR Am J Roentgenol.

[B8] Kass SM, Williams PM, Reamy BV (2007). Pleurisy. Am Fam Physician.

[B9] Longnecker DE, Brown D, Newman M, Zapol WM (2007). Anaesthesiology.

